# A comparison of factors associated with unmet healthcare needs in people with disabilities before and after COVID-19: a nationally representative population-based study

**DOI:** 10.1186/s12913-024-10579-y

**Published:** 2024-01-24

**Authors:** Sujin Lee, Han Nah Park, Hye Jin Nam, Bohye Kim, Ju Young Yoon

**Affiliations:** 1 College of Nursing, Seoul National University, Seoul, Republic of Korea; 2 Research Institute of Nursing Science, Seoul National University, Seoul, Republic of Korea

**Keywords:** Anderson model, COVID-19, Disabled persons, Propensity score matching analysis, Unmet healthcare needs

## Abstract

**Background:**

People with disabilities, who require numerous healthcare services, are vulnerable to unmet healthcare needs. This study aimed to investigate and identify the factors that influence unmet healthcare needs among people with disabilities and to compare these factors before and after the COVID-19 pandemic in South Korea.

**Methods:**

A propensity score matching analysis was conducted using two datasets from the National Survey of Disabled Persons collected in 2017 and 2020. The participants were matched based on variables known to influence healthcare utilization. Based on the Andersen model, logistic regression was performed to analyze the key characteristics of the factors associated with unmet healthcare needs, including predisposing, enabling, and need factors.

**Results:**

Propensity score matching resulted in the inclusion of 1,884 participants in each group: an experimental group and control group. Before COVID-19, factors associated with unmet healthcare needs included sex, age, marital status, and education level (predisposing factors), instrumental activities of daily living dependency, satisfaction with medical staff’s understanding of disability, satisfaction with medical institutional facilities and equipment (enabling factors), subjective health status, and depressive symptoms (need factors). After COVID-19, factors included physical disability, instrumental activities of daily living dependency, and discrimination (enabling factors), and subjective health status, chronic diseases, depressive symptoms, and regular medical care (need factors). No significant predisposing factors affecting unmet healthcare needs were identified after COVID-19.

**Conclusions:**

This study compared the factors affecting unmet healthcare needs among people with disabilities before and after COVID-19. Recognizing the different factors associated with unmet healthcare needs before and after COVID-19, (e.g., sex, type of disability, satisfaction with medical staff’s understanding of disabilities, medical institutional facilities and equipment considering the disabled, discrimination, chronic diseases, and regular medical care) may help governments and policymakers establish strategies to reduce and prevent unmet healthcare needs during and a future crisis.

**Supplementary Information:**

The online version contains supplementary material available at 10.1186/s12913-024-10579-y.

## Background

South Korea’s National Health Insurance aims to guarantee and ensure equitable access to healthcare services regardless of an individual’s health condition or ability to pay [1]. However, although the government claims to offer equitable healthcare services to all citizens, some members of the community still experience limited access to care due to high deductibles, care not covered by insurance services, and a geographical imbalance in the distribution of medical services. In particular, people with disabilities who need a particularly high level of healthcare, have reported that they experienced unmet healthcare needs due to various factors that prevent access to healthcare services [2, 3].

The range of health and healthcare services that individuals with disabilities require varies due to complex social and environmental factors, infrastructure issues, and individual characteristics of people with disabilities. For example, at the societal level, unlike South Korea (Korea hereafter), where the financial burden is the greatest obstacle for people with disabilities, a study conducted in Iran reported that insufficient insurance coverage for medical expenses and low physical accessibility resulted in unmet healthcare needs among adults with disabilities [4]. Chen et al. [5] also identified several individual factors in China impacting unmet healthcare needs in older adults with disabilities including instrumental activities of daily living (IADL) dependency, relationships with caregivers, and monthly income.

Another recent factor has been the COVID-19 disease starting in 2019. COVID-19 spreads through droplets or aerosols, and the main symptoms include fever, malaise, cough, shortness of breath, and pneumonia. However, in patients with impaired immune function or comorbidities, it may cause additional severe complications or even death [6]. During the COVID-19 pandemic, the Korean government implemented a social distancing policy from March 2020 to April 2022 that included avoiding visiting crowded places [7]. These unavoidable measures severely impacted the provision of welfare services [8] including medical services. In particular, the International Disability Alliance (IDA) raised concerns about the insufficient availability of information on infectious diseases and the lack of an appropriate response or support for medical treatment, such as quarantine or screening guidelines [9, 10]. One of the greatest challenges during the COVID-19 pandemic was difficulty using healthcare services among people with disabilities [10]. A nationwide survey of people with disabilities found that approximately 32.4% of the population living with disabilities was not receiving the necessary healthcare services, which was much higher than that in 2017 (17.0%) before the COVID-19 outbreak [11, 12]. A study of 1,500 people with severe disabilities found that 52.9% of participants reported experiencing significant distress as a result of COVID-19 [13]. However, relatively little is known about the impact of COVID-19 on healthcare services and the unmet healthcare needs of people with disabilities. The immense challenges individuals with disabilities faced during the pandemic, including their unmet healthcare needs, underscores the urgency of addressing their unique needs when a global health crisis causes sudden and drastic changes.

“Unmet healthcare needs” is defined as the lack of services that are deemed necessary to avoid adverse health outcomes [14]. Identifying unmet healthcare needs is an important indicator of blind spots in the healthcare system. In particular, access to quality healthcare has a major impact on achieving health equity and is a key to establishing healthy communities [15]. However, when there is a gap healthcare services (i.e., unmet healthcare needs), it is necessary to first examine the factors that affect individuals to identify the vulnerabilities of a specific group, such as people with disabilities. This study applied the Andersen healthcare utilization model, which explains the factors that lead to the use of healthcare services. This conceptual model helps researchers determine the conditions related to healthcare utilization based on three categories: predisposing, enabling, and need factors. Predisposing factors in this model refer to characteristics such as demographic factors that existed prior to the experience of unmet healthcare needs. Enabling factors that affect accessibility to healthcare services include income and personal and family resources. Need factors related to health status include factors that lead individuals to utilize healthcare services [16, 17]. The present study examined and compared these three types of factors affecting unmet healthcare needs among people with disabilities based on the Andersen healthcare utilization model both before and after the start of the COVID-19 pandemic.

Identifying factors that influence unmet healthcare needs is a critical task so medical professionals can provide appropriate and timely healthcare services. It is particularly important to discuss the unmet healthcare needs of people with disabilities who frequently require numerous healthcare services. Any obstacles can lead to the discontinuation of treatment and thus health deterioration. Therefore, this study aimed to analyze and identify what factors affect unmet healthcare needs among people with disabilities and to compare these factors before and after the COVID-19 outbreak in 2019. This study used national data from the National Survey of Disabled Persons in Korea.

## Methods

### Data and participants

This study used two datasets: the 2017 and 2020 National Survey of Disabled Persons by the Ministry of Health and Welfare and the Korea Institute for Health and Social Affairs (KIHASA). The National Survey of Disabled Persons is a nationally representative survey conducted every three years to understand the living conditions and welfare needs of people with disabilities. The 2017 survey was conducted from September to October 2017, and the 2020 survey was conducted from October 2020 to February 2021. In 2017, from a sample of 36,200 households, 6,549 people with disabilities were surveyed through sampling in 250 survey areas. The 2020 survey included 7,025 people with disabilities who were registered in the national database across 248 survey areas without household sampling due to the COVID-19 pandemic. Complete details related to this survey are provided in the disability survey report by the Ministry of Health and Welfare of South Korea [11, 18].

The inclusion criterion for this study was having a physical or intellectual disability, which accounted for the largest proportion of disabilities. According to the enforcement decree of the Act on Welfare of Persons with Disabilities in Korea, a person is defined as having an intellectual disability if they display insufficient or incomplete development of intellectual abilities due to a permanent delay in mental development and have considerable difficulty handling their own work and adapting to social life. A person is defined as having a physical disability if they have one or more of the following conditions: (1) a permanent impairment of the function of one arm, one leg, or the trunk; (2) lost the thumb of one hand above the knuckle joint, or two or more fingers of one hand, including the second finger, above the middle phalange; (3) lost one leg above the Lisfranc joint; (4) lost both big toes; (5) lost the function of the thumb of one hand or of two or more fingers, including the second finger, of one hand; (6) a severely short stature due to dwarfism or marked deformity of the spine; or (7) been recognized as having a disability equivalent to or higher than the degree of disability corresponding to any of the aforementioned items (1–6) [19]. Individuals with disabilities aged < 18 years were excluded from the analysis.

### Measures

#### Dependent variable: unmet healthcare needs

Unmet healthcare needs are defined as a state in which necessary healthcare services are not properly provided despite having a need for such services (i.e., the number of healthcare services that are not met) [14]. In this study, unmet healthcare needs were identified through a survey question asking participants whether they were unable to go to a medical facilith (e.g., hospital, clinic) despite wanting to within the past year. A “yes” answer indicated that they had experienced unmet healthcare needs during the past year.

#### Independent variables

Based on previous studies, the independent variables were divided into predisposing, enabling, and need factors using the Anderson healthcare utilization model [17, 20]. This model has been extensively used to examine the factors involved in the utilization of healthcare services [21] and considers both internal and external factors affecting utilization. Predisposing factors (i.e., individual characteristics) include sex, age, marital status, and education level. Enabling factors include access to healthcare services, including the type and degree of disability, monthly income, working status, national basic livelihood, activities of daily living (ADL), IADL dependency, ability to go out alone, vehicle ownership, disability discrimination experience, satisfaction with medical staff’s understanding of their disability, satisfaction with communication with medical staff, and satisfaction with medical institutions and equipment. Need factors are subjective health conditions that trigger the use of healthcare services, including the presence or absence of chronic diseases, depressive symptoms, and regular medical care.

### Statistical analysis

In this study, SAS 9.4 (SAS Institute, Cary, NC, USA) and IBM SPSS 23.0 (IBM Corp., Armonk, NY, USA) software were used to analyze and compare the factors affecting unmet health care needs for people with intellectual and physical disabilities before and after the COVID-19 outbreak. First, propensity score matching analysis was applied to reduce selection bias between the variables of the experimental and control groups. In this study, the nearest-neighbor matching method and caliper matching were mixed and applied. A 1:1 matching method and a caliper range of 0.01 were applied, and a paired *t*-test was performed to verify the results of the propensity score matching. Additionally, the visual data of the propensity score and standardized mean difference, calculated using the propensity score mean difference and standard deviation, were graphically compared [22]. In the propensity score matching analysis, the dependent variable was the COVID-19 outbreak period (from 2019 to 2020). Based on previous studies, we selected eight independent variables: sex, age, marital status, education level, disability type, education level, residence, and monthly income [23–25].

Second, descriptive statistical analysis was conducted including the means and standard deviation, frequency, and percentage, Chi-square tests, and independent-sample *t*-tests were conducted to understand the demographic and sociological characteristics of the propensity score-matched data before and after the COVID-19 outbreak.

Finally, logistic regression analysis was performed to compare and analyze the factors affecting the unmet health care needs of people with disabilities before and after COVID-19. Additional logistic regression analysis was also performed to compare physical and intellectual disabilities since factors affecting unmet healthcare needs may vary depending on the type of disability.

## Results

### Propensity score matching estimation results

**Fig. 1:**
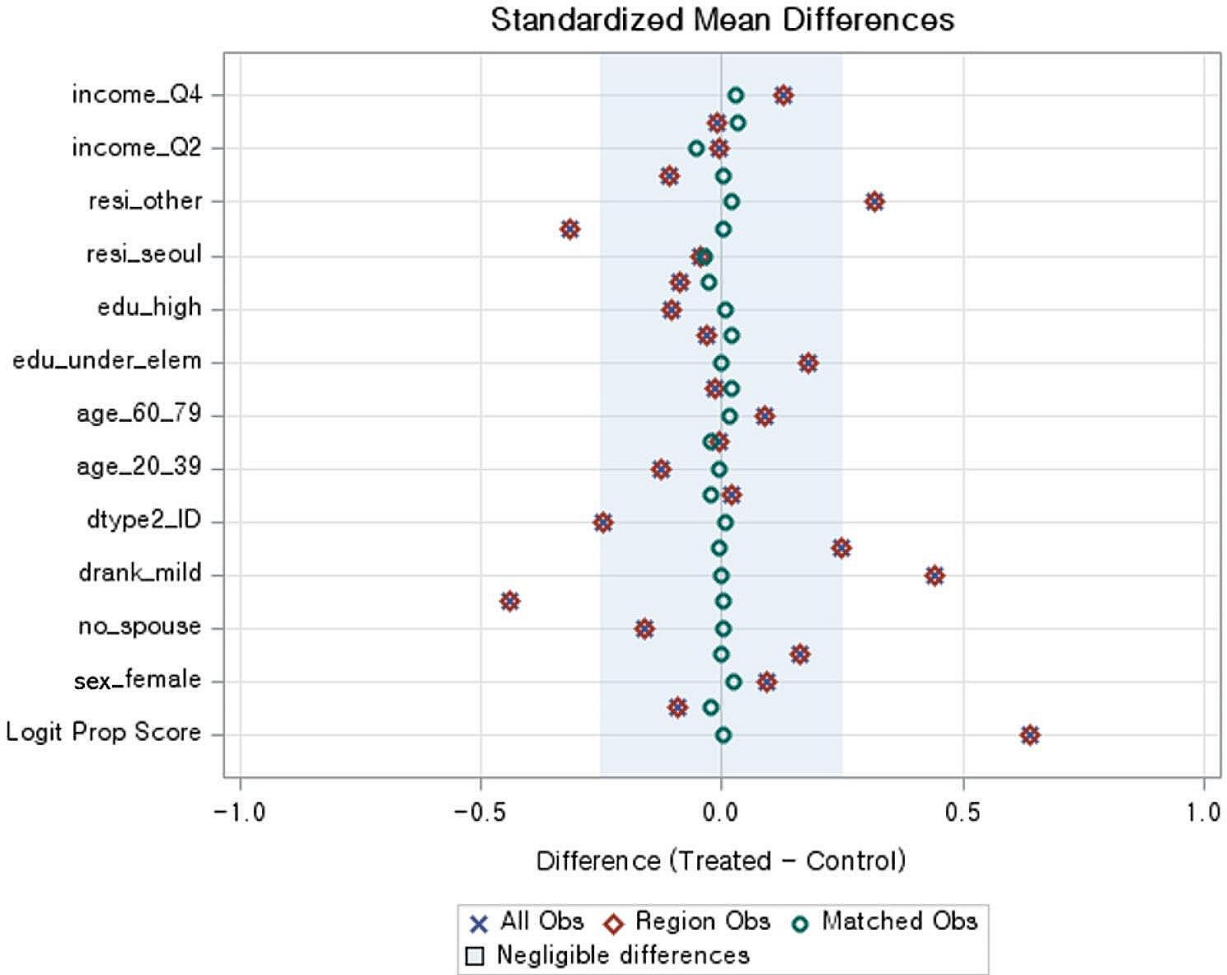
Standardized mean differences of covariates for the matched and unmatched samples

**Fig. 2:**
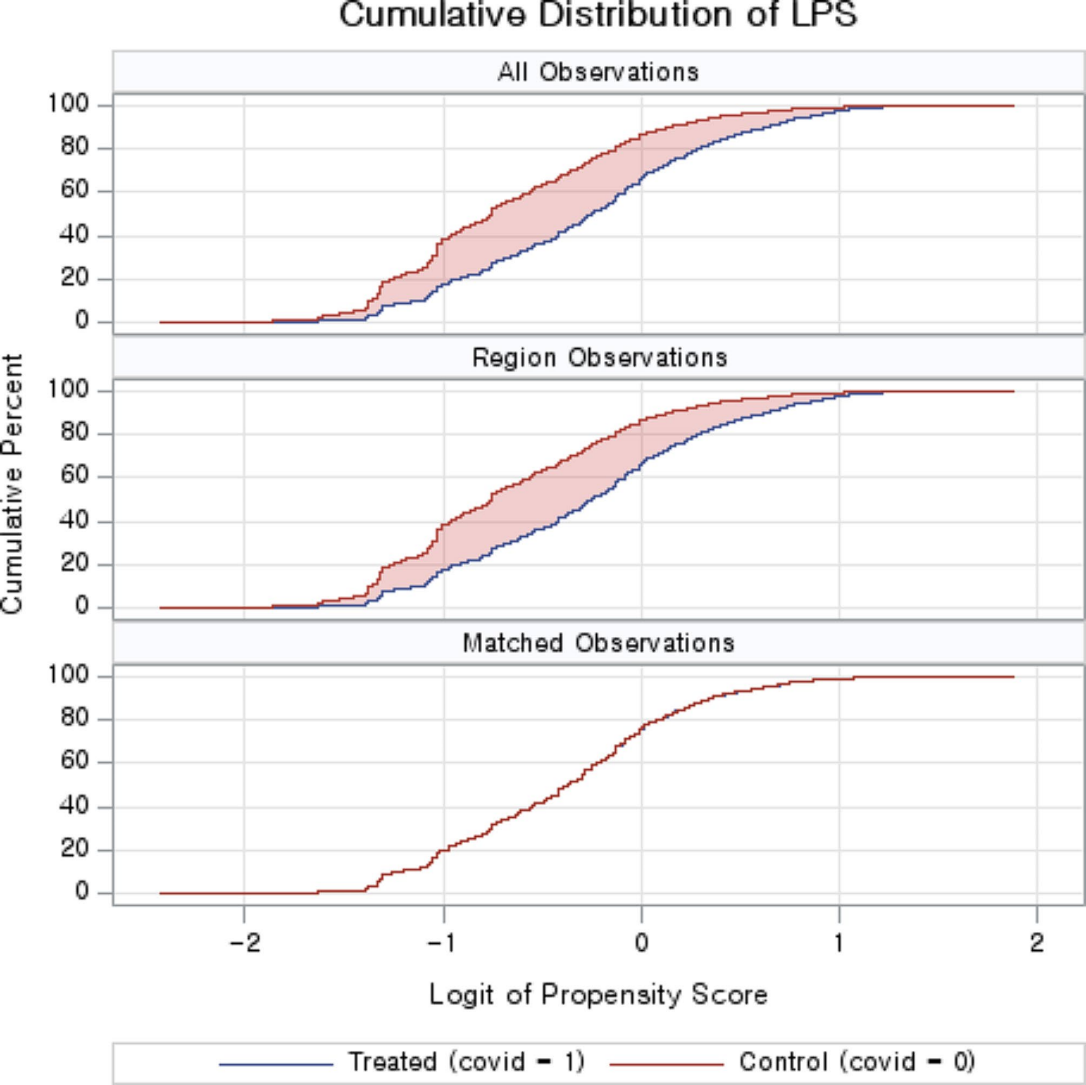
Cumulative distribution of logit of propensity scores of the matched and unmatched samples (LPS)

Based on the type of disability and age, 3,534 people with disabilities were included in the propensity score matching before the COVID-19 outbreak (control group) and 2,192 were included after the outbreak (experimental group). After the propensity score matching process, the experimental group and control group included 1,884 participants in each group (Table 1). To verify the results of the propensity score matching, a standardized difference diagram was used to confirm that the average difference in all variables after matching was close to zero (Fig. 1). A difference between the control and experimental groups was noted in the cumulative distribution of propensity scores before matching, whereas the distribution of the control and experimental groups were almost identical after matching (Fig. 2).

**Table 1:** Characteristics before and after propensity score matching

(UNIT: n (%))									
Variables	Categories	Before Propensity Score Matching (*n* = 5,726)				After Propensity Score Matching (*n* = 3,768)			
		Before COVID-19 outbreak (*n* = 3,534)	After COVID-19 outbreak (*n* = 2,192)	*t*	*p*	Before COVID-19 outbreak (*n* = 1,884)	After COVID-19 outbreak (*n* = 1,884)	*t*	*p*
Sex	Male	1,909 (56.3)	1,286 (56.9)	–4.10	< 0.001	1,084 (58.7)	1,105 (56.9)	–0.70	0.482
	Female	1,625 (43.7)	906 (43.1)	4.10	< 0.001	800 (41.3)	779 (43.1)	0.70	0.482
Age (years)	19	23 (0.8)	11 (0.2)	–0.22	0.827	6 (0.4)	9 (0.2)	–0.78	0.439
	20–39	304 (10.0)	273 (10.2)	–4.19	< 0.001	203 (12.7)	207 (9.2)	–0.21	0.833
	40–59	1,071 (32.6)	671 (30.2)	–1.26	0.206	584 (32.4)	603 (31.1)	–0.66	0.511
	60–79	1,749 (47.4)	986 (45.9)	3.83	< 0.001	877 (45.0)	864 (46.7)	0.42	0.674
	≥ 80	387 (9.3)	251 (13.4)	–0.14	0.886	214 (9.6)	201 (12.8)	0.68	0.499
Residential area	Seoul	319 (14.7)	226 (14.3)	–1.55	0.121	170 (14.3)	191 (14.1)	–1.15	0.250
	Metropolitan	669 (23.4)	714 (23.1)	–15.47	< 0.001	511 (32.5)	509 (20.0)	0.07	0.944
	Others^a^	2,546 (61.9)	1,252 (62.6)	14.61	< 0.001	1,203 (53.2)	1,184 (65.9)	0.63	0.531
Monthly income (10,000 KRW)	< 100	912 (20.5)	674 (31.3)	–2.01	0.044	555 (24.4)	552 (30.3)	0.11	0.914
	100–<200	1,006 (26.7)	632 (26.9)	0.40	0.691	514 (25.3)	561 (27.6)	–1.66	0.096
	200–<300	572 (17.0)	362 (17.5)	–1.45	0.148	325 (18.0)	303 (17.3)	0.97	0.330
	≥ 300	1,044 (35.9)	524 (24.3)	2.89	0.004	490 (32.3)	468 (24.8)	0.82	0.414
Education	≤Elementary school	1,604 (38.3)	801 (36.3)	6.67	< 0.001	712 (31.3)	715 (37.0)	–0.10	0.918
	Middle school	597 (17.7)	398 (19.0)	–0.86	0.391	352 (19.1)	338 (19.0)	0.60	0.547
	High school	947 (30.7)	691 (30.9)	–4.24	< 0.001	590 (34.0)	584 (30.6)	0.21	0.833
	≥College	386 (13.3)	302 (13.7)	–3.24	0.001	230 (15.7)	247 (13.4)	–0.82	0.413
Degree of disability	Mild (grade 4–6)	2,585 (72.1)	1,146 (70.2)	14.05	< 0.001	1,109 (58.4)	1,110 (74.9)	–0.03	0.973
	Severe (grade 1–3)	949 (27.9)	1,046 (29.8)	–14.05	< 0.001	775 (41.6)	774 (25.1)	0.03	0.973
Type of disability	Physical	3,147 (88.6)	1,759 (87.4)	7.70	< 0.001	1,569 (83.0)	1,573 (89.6)	–0.18	0.859
	Intellectual	387 (11.4)	433 (12.6)	–7.67	< 0.001	315 (17.0)	311 (10.4)	0.18	0.859
Spouse	Yes	2,001(55.8)	1,065 (52.3)	4.86	< 0.001	962 (50.8)	964 (54.1)	–0.07	0.948
	No^b^	1,533(44.2)	1,127 (47.7)	–4.86	< 0.001	922 (49.2)	920 (45.9)	0.07	0.948

Note. n = unweighted, %=weighted; COVID = corona virus disease; KRW = Korean won; ^a^ Sejong, 8 provinces, and Jeju-do; ^b^No spouse = Widowed, Divorced, Separated, Never married

### Comparison of key variable characteristics before and after COVID-19

Data analysis after the propensity scores matching showed that the number of people with disabilities who experienced unmet health care needs after the COVID-19 outbreak increased by approximately 1.7 times, from 360 to 604 (Appendix 1). People experienced different variables caused differences in unmet healthcare needs before and after the COVID-19 outbreak were different based on key variable characteristics. Specifically, before COVID-19, participants reported different satisfaction levels with healthcare services based the presence or absence of a spouse (χ² = 4.37, *p* = 0.037) and the degree to which participants considered their disability to be well understood by medical staff (*t* = − 2.38, *p* = 0.018). These factors were not observed after the COVID-19 outbreak. However, there was no statistically significant difference in unmet healthcare needs before and after COVID-19 based on the following factors: the type of disability (χ² = 4.36, *p* = 0.037), whether the people with disabilities were National Basic Livelihood recipients (χ² = 7.80, *p* = 0.005), the degree of discrimination against people with disabilities (*t* = − 4.95, *p* < 0.001), and the presence or absence of chronic diseases (χ² = 57.38, *p* < 0.001) (Table 2).

**Table 2:** Differences in participants’ characteristics between unmet and met healthcare needs before and after COVID-19

Variables	Categories or range	Before COVID-19 outbreak (*n* = 1,884)		χ^2^ or t	*p*	After COVID-19 outbreak (*n* = 1,884)		χ^2^ or t	*p*
		Unmet healthcare needs (*n* = 360)	Met healthcare needs (*n* = 1,524)			Unmet healthcare needs (*n* = 604)	Met healthcare needs (*n* = 1,280)		
		n (%) or Mean ± SD				n (%) or Mean ± SD			
**Predisposing factors**									
Sex	Male	170 (47.2)	914 (60.0)	19.38	< 0.001	318 (52.6)	787 (61.5)	13.21	< 0.001
	Female	190 (52.8)	610 (40.0)			286 (47.4)	493 (38.5)		
Age (years)		62.39 ± 16.12	60.48 ± 16.34	2.00	0.046	62.67 ± 15.20	59.42 ± 16.31	–4.23	< 0.001
Spouse	Yes	166 (46.1)	796 (52.2)	4.37	0.037	293 (48.5)	671 (52.4)	2.51	0.113
	No^a^	194 (53.9)	728 (47.8)			311 (51.5)	609 (47.6)		
Education	≤Elementary school	153 (42.5)	559 (36.7)	7.88	0.049	278 (46.0)	437 (34.1)	27.30	< 0.001
	Middle school	74 (20.6)	278 (18.2)			106 (17.5)	232 (18.1)		
	High school	96 (26.7)	494 (32.4)			156 (25.8)	428 (33.4)		
	≥College	37 (10.3)	193 (12.7)			64 (10.6)	183 (14.3)		
**Enabling factors**									
Type of disability	Physical disability	306 (85.0)	1,263 (82.9)	0.95	0.331	520 (86.1)	1,053 (82.3)	4.36	0.037
	Intellectual disability	54 (15.0)	261 (17.1)			84 (13.9)	227 (17.7)		
Degree of disability	Mild (grade 4–6)	212 (58.9)	897 (58.9)	0.01	0.992	342 (56.6)	768 (60.0)	1.93	0.164
	Severe (grade 1–3)	148 (41.1)	627 (41.1)			262 (43.4)	512 (40.0)		
Monthly household income (10,000 KRW)		191.97 ± 172.13	226.77 ± 189.34	–3.38	0.001	181.51 ± 165.83	214.85 ± 205.19	3.49	< 0.001
Employment status	Employed	120 (33.3)	641 (42.1)	9.21	0.002	173 (28.6)	476 (37.2)	13.27	< 0.001
	Unemployed	240 (66.7)	883 (57.9)			431 (71.4)	804 (62.8)		
National Basic Livelihood	Beneficiary	77 (21.4)	299 (19.6)	0.57	0.450	192 (31.8)	328 (25.6)	7.80	0.005
	Non-beneficiary	283 (78.6)	1,225 (80.4)			412 (68.2)	952 (74.4)		
ADL dependency	1–4^b^	1.25 ± 0.48	1.14 ± 0.33	4.06	< 0.001	1.30 ± 0.62	1.15 ± 0.46	–5.14	< 0.001
IADL dependency	1–4^b^	1.60 ± 0.80	1.40 ± 0.66	4.29	< 0.001	1.65 ± 0.87	1.41 ± 0.76	–5.87	< 0.001
Going outdoors independently	Yes	301 (83.6)	1,357 (89.0)	8.14	0.004	469 (77.6)	1,083 (84.6)	13.70	< 0.001
	No	59 (16.4)	167 (11.0)			135 (22.4)	197 (15.4)		
Owned a car	Yes	145 (40.3)	778 (51.0)	13.52	< 0.001	262 (43.4)	686 (53.6)	17.13	< 0.001
	No	215 (59.7)	746 (49.0)			342 (56.6)	594 (46.4)		
Experience of discrimination	1–4^c^	1.91 ± 0.82	1.90 ± 0.77	0.04	0.965	2.27 ± 0.72	2.10 ± 0.69	–4.95	< 0.001
Satisfaction with medical staff’s understanding of disability	1–5^d^	3.40 ± 0.97	3.53 ± 0.84	–2.38	0.018	3.60 ± 0.72	3.60 ± 0.69	0.09	0.926
Satisfaction with communication with medical staff	1–5^d^	3.71 ± 0.69	3.64 ± 0.68	1.93	0.054	3.65 ± 0.73	3.66 ± 0.68	0.44	0.658
Satisfaction with medical institution facilities and equipment	1–5^d^	3.59 ± 0.69	3.48 ± 0.71	2.67	0.008	3.50 ± 0.72	3.59 ± 0.61	2.43	0.015
**Need factors**									
Subjective health status	1–5^e^	3.73 ± 0.91	3.31 ± 0.87	8.26	< 0.001	3.62 ± 0.91	3.29 ± 0.87	–7.49	< 0.001
Chronic disease	Yes	289 (80.3)	1,149 (75.4)	3.84	0.050	475 (78.6)	781 (61.0)	57.38	< 0.001
	No	71 (19.7)	375 (24.6)			129 (21.4)	499 (39.0)		
Depressive symptoms	Yes	92 (25.6)	184 (12.1)	42.33	< 0.001	162 (26.8)	140 (10.9)	76.92	< 0.001
	No	268 (74.4)	1,340 (87.9)			442 (73.2)	1,140 (89.1)		
Regular medical care	Yes	288 (80.0)	1,193 (78.3)	0.51	0.474	456 (75.5)	913 (71.3)	3.59	0.058
	No	72 (20.0)	331 (21.7)			148 (24.5)	367 (28.7)		

Note. n, %=unweighted; COVID = corona virus disease; KRW = Korean won; SD = Standard Deviation; ADL = activities of daily living; IADL = instrumental activities of daily living; ^a^No spouse = Widowed, Divorced, Separated, Never married; ^b^High scores indicate that more support is needed for ADL or IADL; ^c^High scores indicate more experiences of discrimination; ^d^High scores indicate higher satisfaction; ^e^High scores indicate worse subjective health status

### Factors affecting unmet healthcare needs for people with disabilities

Models representing the two time periods (before and after the COVID-19 outbreak) were constructed and analyzed to compare the factors affecting the unmet healthcare experience of people with disabilities before and after the COVID-19 outbreak. In both models, the variance expansion factor value was < 10, indicating that the possibility of multicollinearity was low. The suitability of the regression analysis in the pre- and post-COVID-19 models was statistically significant.

Table 3 shows the analysis results of the factors affecting unmet healthcare needs among people with disabilities. The analysis of the pre-COVID-19 model indicated that among the predisposing factors, women with disabilities (odds ratio [OR] = 1.50, *p* < 0.001) reported more unmet healthcare needs than men; all other variables had no significant effect. Among the enabling factors, more unmet healthcare needs were reported when the IADL score was high (OR = 1.07, *p* = 0.003), indicating that the subject was more dependent on IADL. In addition, the lower the satisfaction with the medical staff’s understanding of their disability (OR = 0.84, *p* = 0.042), the more unmet healthcare needs were reported. Among the need factors, unmet healthcare needs were reported when participants experienced worse subjective health status (OR = 0.62, *p* < 0.001) and depressive symptoms (OR = 1.69, *p* = 0.001).

**Table 3:** Factors associated with unmet healthcare needs before and after COVID-19

Variables	Categories	Before COVID-19					After COVID-19				
		B	S.E.	OR	95% CI	*p*	B	S.E.	OR	95% CI	*p*
**Predisposing Factors**											
Sex	Female	0.404	0.14	1.50	1.15–1.96	< 0.001	0.167	0.12	1.18	0.94–1.49	0.155
	(ref. Male)										
Age		–0.007	0.01	0.99	0.98–1.01	0.316	–0.004	0.01	0.99	0.98–1.01	0.443
Spouse	No	–0.001	0.14	0.99	0.76–1.31	0.995	0.046	0.12	1.05	0.83–1.32	0.702
	(ref. Yes)										
Education	≤Elementary school	–0.139	0.26	0.87	0.52–1.46	0.596	0.223	0.22	1.25	0.82–1.92	0.308
	Middle school	0.078	0.25	1.08	0.66–1.77	0.759	0.092	0.22	1.10	0.72–1.68	0.670
	High school	–0.146	0.23	0.86	0.55–1.35	0.523	–0.067	0.19	0.94	0.65–1.36	0.723
	(ref. ≥College)										
**Enabling Factors**											
Type of disability	Physical disability	0.391	0.29	1.48	0.83–2.63	0.183	0.479	0.23	1.62	1.03–2.54	0.038
	(ref. Intellectual disability)										
Degree of disability	Severe	–0.120	0.16	0.89	0.65–1.20	0.440	0.094	0.14	1.10	0.84–1.43	0.485
	(ref. Mild)										
Monthly household income		–0.001	0.00	1.00	0.99–1.00	0.190	0.000	0.00	1.00	0.99–1.00	0.801
Employment status	Employed	–0.107	0.15	0.90	0.67–1.21	0.486	–0.162	0.14	0.85	0.65–1.11	0.232
	(ref. Unemployed)										
National basic livelihood	Beneficiary	–0.190	0.17	0.83	0.59–1.16	0.272	–0.007	0.14	0.99	0.76–1.30	0.959
	(ref. Non-beneficiary)										
ADL dependency		–0.008	0.02	0.99	0.95–1.03	0.719	0.001	0.01	1.00	0.98–1.03	0.916
IADL dependency		0.065	0.02	1.07	1.02–1.12	0.003	0.043	0.02	1.04	1.01–1.08	0.004
Going outdoors independently	Yes	0.376	0.25	1.46	0.89–2.40	0.139	0.218	0.19	1.24	0.86–1.80	0.248
	(ref. No)										
Owned a car	Yes	–0.281	0.15	0.76	0.56–1.02	0.063	–0.120	0.13	0.89	0.69–1.15	0.357
	(ref. No)										
Experience of discrimination		–0.147	0.09	0.86	0.73–1.02	0.082	0.277	0.08	1.32	1.12–1.55	0.001
Satisfaction with medical staff’s understanding of disability		–0.174	0.09	0.84	0.71–0.99	0.042	0.018	0.10	1.02	0.83–1.24	0.856
Satisfaction with communication with medical staff		0.049	0.11	1.05	0.85–1.31	0.661	0.094	0.10	1.10	0.90–1.34	0.362
Satisfaction with medical institution facilities and equipment		–0.196	0.10	0.82	0.68–0.99	0.043	–0.167	0.10	0.85	0.70–1.02	0.082
**Need Factors**											
Subjective health status		–0.479	0.09	0.62	0.52–0.74	< 0.001	–0.156	0.07	0.86	0.74–0.99	0.031
Chronic disease	Yes	0.002	0.22	1.00	0.66–1.54	0.992	1.043	0.16	2.84	2.08–3.87	< 0.001
	(ref. No)										
Depressive symptoms	Yes	0.525	0.16	1.69	1.24–2.31	0.001	0.778	0.14	2.18	1.65–2.87	< 0.001
	(ref. No)										
Regular medical care	Yes	–0.306	0.21	0.74	0.49–1.12	0.151	–0.692	0.16	0.50	0.37–0.68	< 0.001
	(ref. No)										
**-2 Log likelihood**		**1,699.05**					**2,161.59**				
**Nagelkerke** ***R*** **²**		**0.11**					**0.14**				
**Hosmer–Lemeshow test**		**χ² = 6.57**, ***p*** **= 0.583**					**χ² = 3.77**, ***p*** **= 0.877**				

Note. ADL = activities of daily living; B = unstandardized coefficient; CI = confidence interval; COVID = corona virus disease; IADL = instrumental activities of daily living; OR = odds ratio; ref.=reference; S.E.=standard error

The post-COVID-19 analysis showed that among the predisposing factors, no variables affected the unmet healthcare needs of people with disabilities. Among the enabling factors, people with physical disabilities (OR = 1.62, *p* = 0.038) had more unmet healthcare needs than those with intellectual disabilities, confirming that there was a difference in unmet healthcare experiences depending on the type of disability after COVID-19. Similar to before the COVID-19 outbreak, unmet healthcare needs occurred when the IADL score was high (OR = 1.04, *p* = 0.004) after COVID-19. In addition, the higher the level of discrimination experienced by the people with disabilities (OR = 1.32, *p* = 0.001), the more unmet healthcare needs were reported. However, discrimination was not an influential variable prior to the COVID-19 outbreak. Conversely, satisfaction with medical staff’s understanding of their disability or medical institution facilities and equipment, did not significantly affect the unmet healthcare experience of people with disabilities after COVID-19, but it had a significant effect before COVID-19.

Another key finding is that all need factors were found to have an effect after the outbreak. In contrast, before the COVID-19 outbreak, only worse subjective health status (OR = 0.86, *p* = 0.031) and the presence or absence of depressive symptoms affected the unmet healthcare needs of people with disabilities. After the pandemic, a worse subjective health condition (OR = 0.86, *p* = 0.031) was associated with high unmet healthcare needs. In addition, unmet healthcare needs were high when the participants had chronic diseases (OR = 2.84, *p* < 0.001) or depressive symptoms (OR = 2.18, *p* < 0.001). Regular medical care (OR = 0.50, *p* < 0.001) also had a negative effect on unmet healthcare needs. This finding demonstrates that continuous healthcare for people with disabilities is an important factor for receiving sufficient healthcare services.

Logistic regression analysis was also conducted to identify factors affecting the unmet healthcare needs of people with physical (Appendix 2) and intellectual disabilities (Appendix 3). Statistically significant factors included having a physical disability, sex, age, IADL dependency, satisfaction with medical institution facilities and equipment, subjective health status, and depressive symptoms. Women with disabilities (OR = 1.555, *p* = 0.005) had more unmet healthcare needs than men. Additionally, if the scores for IADL (OR = 1.084, *p* = 0.007) and depressive symptoms (OR = 1.747, *p* = 0.001) were high, more healthcare needs were unmet. In contrast, older age (OR = 0.984, *p* = 0.034), low satisfaction with medical institution facilities and equipment (OR = 0.777, *p* = 0.016) and better subjective health status (OR = 0.554, *p* < 0.001) were associated with fewer unmet healthcare needs. The more they experienced discrimination, the fewer unmet healthcare needs they reported (OR = 0.495, *p* = 0.007).

## Discussion

This study examined and compared the factors influencing unmet healthcare needs among people with disabilities before and after the COVID-19 pandemic, using national survey data collected in 2017 (before the pandemic) and 2020 (after the pandemic). The results indicated that factors associated with unmet healthcare needs before the outbreak of COVID-19 included being female, having higher IADL scores, a poorer understanding of disabilities by the medical staff, dissatisfaction with the medical facilities and equipment, poorer subjective health status, and depressive symptoms. The factors associated with unmet healthcare needs after the COVID-19 outbreak included physical disability, higher IADL scores, not receiving regular medical treatment, experiencing discrimination against their disability, chronic disease, poor subjective health status, and depressive symptoms.

Overall, our study identified some differences in the factors related to unmet medical needs before and after the COVID-19 outbreak. First, after propensity score matching, the number of people with disabilities who experienced unmet healthcare needs increased from 360 (before the outbreak in 2017) to 604 (after the COVID-19 outbreak in 2020). Similarly, a study in the United States found that adults with disabilities were 1.78 times more likely to experience unmet or delayed healthcare needs compared to those without disabilities during the COVID-19 pandemic [26]. The Korea Health Panel, which investigated Koreans’ unmet healthcare needs in 2020, also found that the rate of unmet healthcare needs among people living with disabilities was 32.4%, which was twice as high as the unmet healthcare needs of people without disabilities (15.1%) [12, 27]. Another study found that 17.0% of people with disabilities experienced unmet healthcare needs in 2017 compared to 8.8% of people without disabilities in 2016 [5]. These results confirm that people with disabilities have more difficulties accessing healthcare services [28]. Moreover, these difficulties increased after the outbreak of COVID-19. During COVID-19, health and medical resources were used intensively for patients with the infectious disease. In addition, many people experienced decreased incomes decreases due to restrictions on economic activities. Social distancing or quarantine and anxiety about the risk of COVID-19 infection in medical institutions were also mentioned as some of the additional reasons for avoiding or delaying treatment [27].

Based on the Andersen model, being female was the only predisposing factor that affected unmet healthcare needs before the COVID-19 outbreak. A systematic literature review on unmet healthcare needs in South Korea and Iran also reported that women had higher unmet healthcare needs due to housework or childcare [29, 30]. Jeon [31] also found that women with disabilities had more difficulty than men utilizing various healthcare services, including health checkups, outpatient treatment, and hospitalizations. Another study in a South Korean city at the beginning of the COVID-19 pandemic confirmed that women experienced unmet healthcare needs more than men [32]. Contrary to the results of these previous studies [29–32] that consistently identified women with disabilities as an influencing factor for unmet healthcare needs, the present study found that a higher number of both men and women experienced unmet healthcare needs after the COVID-19 outbreak. However, the number was not statistically significant, which is similar to the findings of a study that reported no statistically significant relationships between sex and unmet healthcare needs [33]. On the other hand, Khattar et al. [33] found a significant increase in two other influencing factors—depression and anxiety—among both men and women during the COVID-19 pandemic. Thus, consideration of the demand for unmet healthcare among people with disabilities should include the characteristics of the COVID-19 pandemic.

Among the enabling factors, our study confirmed that people with physical disabilities experienced unmet healthcare needs approximately 1.6 times more than those with intellectual disabilities after the outbreak of COVID-19. A 2019 study in China also reported that although the gap by type of disability was not large, people with physical disabilities experienced the most unmet healthcare needs [34]. However, in a study conducted in the United States, people with intellectual disabilities experienced more unmet healthcare needs than those with physical disabilities [26]. In the current study, the type of disability was not a major factor before the COVID-19 outbreak. However, it can be assumed that while strict social distancing was enforced due to the COVID-19 outbreak, people with physical disabilities were more likely to experience greater difficulty accessing healthcare services than those with intellectual disabilities due to their functional limitations.

Discrimination was another significant factor affecting unmet healthcare needs after the COVID-19 outbreak. Several previous studies have demonstrated a strong correlation between unmet healthcare needs and discrimination or avoidance by medical staff. Among people with disabilities who experienced discrimination [35, 36], 30% subsequently developed new or worsening disabilities [37]. A recent in-depth study that identified unmet healthcare needs of people with disabilities during the COVID-19 pandemic found that discrimination against those with disabilities did not increase significantly during the pandemic [38]. Rather, people with disabilities were more burdened by a combination of disability stigma and the pandemic. Discrimination and stigma against people with disabilities can infringe on their right to quality healthcare. The findings of the current study indicated that before the COVID-19 outbreak, the level of satisfaction with facilities and equipment of healthcare services, as well as with the medical staff’s understanding of disability was associated with the unmet healthcare needs of people with disabilities. Both discrimination and facility- or equipment-related factors represent the characteristics of social support. In other words, when welfare services that support mobility or transport systems for people with disabilities were readily available before the COVID-19 outbreak, only the quality of the medical system affected unmet healthcare needs. However, after the pandemic, discrimination against people with disabilities became a stronger factor in unmet healthcare needs due to inequitable disaster support for people with disabilities caused by a lack of mobility support services, information related to COVID-19, and the limited supply of equipment to prevent infectious diseases [38]. The IDA advised governments and communities to take appropriate action as discrimination against people with disabilities may have intensified during the COVID-19 crisis [39]. To address this need, the World Health Organization and the Office of the High Commissioner for Human Rights (OHCHR) published recommendations for people with disabilities [40, 41].

Among the enabling factors, IADL dependency influenced unmet healthcare needs both before and after the COVID-19 outbreak. The IADL score is the degree of independent activity performance, and higher scores reflect greater dependency. IADL is different from ADL. IADL, which reflects an individual’s self-care ability, was found to have a significant effect on unmet healthcare needs. For example, the questionnaire used in this study to assess IADL included questions related to their ability to manage medications, which is related to the use of healthcare services [42]. A previous study also indicated that individuals who require assistance with IADL but not with ADL are at higher risk of not having access to the assistive devices they need to utilize healthcare services [43]. Therefore, the ability to perform IADL can have a greater impact on an individual’s access to healthcare services compared to the ability to perform ADL.

In terms of the impact of dependent IADL on unmet healthcare needs both before and after the COVID-19 outbreak, the findings revealed insufficient continuous support for the independence of people with disabilities. Consequently, to resolve the healthcare needs of people with disabilities, healthcare professionals should actively discuss and support people with disabilities so they can continue independent activities. They should also reevaluate the effectiveness of the existing welfare system for daily support for this population.

Among the need factors, poor subjective health status and depressive symptoms led to an increase in unmet healthcare needs both before and after COVID-19. This result is similar to previous studies establishing these two variables as well-known factors that affect the unmet healthcare and dental care needs of people with and without disabilities [44–46]. This study also revealed that subjective health status had a greater impact before COVID-19, whereas depressive symptoms had a greater impact on unmet healthcare needs after the COVID-19 outbreak. Subjective health status includes social, psychological, and physical health needs. Thus, given the significant impact on unmet healthcare needs among people with disabilities, healthcare professionals and policymakers must reconsider the overall quality of life of people with disabilities. Furthermore, since self-assessed changes in health (SACH) are a more valid indicator to predict health status than one-time subjective health status, SACH items should be included in national surveys related to people with disabilities. Another consideration should be depression in this group. Studies by Kim et al. [47] and Adams et al. [48] showed that the group with depression reported higher unmet healthcare needs after COVID-19 than before the pandemic, and that an increase in depression due to quarantine after COVID-19 infection may have affected unmet healthcare needs.

Among the four need factors, other than the variables that were affected both before and after the COVID-19, chronic disease was a significant factor for unmet healthcare needs only after the outbreak. Since people with disabilities who have chronic diseases needed to continue using healthcare services, they were at risk of unmet healthcare needs during the COVID-19 pandemic. For example, a previous study on patients with hypertension and diabetes reported that one in six patients experienced unmet healthcare needs during the COVID-19 pandemic, which was a significantly higher rate than in those without chronic diseases [49]. A Canadian study of the adult people without disabilities also confirmed that people with chronic diseases experienced difficulty going to the hospital or seeing a doctor during the COVID-19 pandemic, and expressed concerns about their future health [50]. It is likely that those with a disability and chronic diseases faced even more challenges and unmet needs than those without a disability.

In the current study, regular medical care was another significant factor associated with unmet healthcare needs after the COVID-19 outbreak. People with disabilities who regularly received medical care before the pandemic were less likely to experience unmet healthcare needs. Regular medical treatment, as a need factor, implies frequent use and thus high acceptance of healthcare services. Community medical and healthcare service providers should provide follow-up care and case-based management because people with disabilities need continuous and comprehensive healthcare based on the varied needs, type, and degree of their disability. To achieve the continuous use of healthcare services, Korea has introduced a “primary care team for people with disabilities,” which includes visiting medical and nursing care. However, this service is still insufficient [11, 51]. Establishing and expanding this system will provide regular and systematic healthcare services for community-dwelling individuals living with disabilities.

### Limitations

Despite the important findings of our study, there are several limitations. First, the concept of measuring unmet healthcare needs may vary depending on how healthcare services are defined. The data used in this study defined unmet healthcare needs as inaccessibility to hospitals/clinics and did not include unmet healthcare needs related to other access such as on-site treatment or local community services. For example, in Korea, a visiting care project called “Primary Care for People with Disabilities” provides community health care services to people with chronic diseases or people with severe disabilities who have lived in residential facilities and then moved to the community. However, the project is still in the pilot operation stage and the services are insufficient for the wider population. Thus, they were not included in this study. In addition, the perception of unmet healthcare needs may be interpreted differently based on one’s medical knowledge, but we were unable to measure participants’ knowledge.

Another limitation is that the data were based on subjective perceptions of unmet healthcare needs, but individuals’ perceptions of their level of these needs may vary. In addition, due to the use of the same questionnaire developed before the COVID-19 outbreak, including specific variables related to unmet healthcare needs during the COVID-19 pandemic in the analysis was not possible. Fourth, since this study used existing data, we were unable to analyze the types and content of the healthcare services. Given that unmet health care needs can vary depending on the type and content of the healthcare service, future research should consider these variables. Fifth, propensity score matching can cause overmatching bias. To minimize this bias, a more careful approach in future studies is needed to select the propensity score matching variables. We expect that follow-up studies will identify and discuss more comprehensive aspects of unmet healthcare needs considering the unique characteristics and environments of people with disabilities. For example, the needs and difficulties may vary depending on the characteristics of each disability. Therefore, follow-up studies should aim to identify factors for each disability. Moreover, in the post-COVID-19 era, follow-up studies should trace what factors continue to influence unmet healthcare needs so the rights of people with disabilities to healthcare can be guaranteed.

Despite these limitations, compared to previous studies using cross-sectional data, our study is significant in comparing and explaining the cumulative risk of unmet healthcare needs before and during the prolonged pandemic using a regular national survey. In particular, adjusting the samples using propensity score matching helped us identify and compare changes in the influencing factors associated with unmet healthcare needs before and after the COVID-19 outbreak. The results of this study could serve as the basis for customized strategies rather than an extension of existing healthcare services in disaster situations such as infectious disease epidemics. This study also confirmed that people with disabilities who were dependent (a high IADL level) had poor subjective health, and that depressive symptoms had a greater impact on unmet healthcare needs. This study highlights the need to pay more attention to this vulnerable group of disabled individuals, especially those who have difficulty accessing healthcare.

## Conclusion

The current study identified several factors affecting unmet healthcare needs before and after the COVID-19 outbreak that began in 2019, when the same conditions were set using propensity score matching. Common factors influencing unmet health healthcare needs both before and after COVID-19 included high IADL dependency, poor subjective health status, and depressive symptoms. The factors affecting unmet healthcare needs before COVID-19 in 2017 were female and satisfaction with medical staff’s understanding of their disability and with the facilities and equipment in healthcare services. However, factors affecting unmet healthcare needs after the outbreak of COVID-19 in 2020 included having a physical disability that was not an intellectual disability, experiencing discrimination against people with disabilities, having chronic diseases, and participating in regular medical treatment. Therefore, in the pandemic, which required extensive limitations on social activities, a different customized strategy should have been provided so people with disabilities could obtain appropriate and adequate healthcare services. More attention and support should be given to the factors that affect unmet healthcare needs even after the pandemic to enhance and maintain access to healthcare services.

### Electronic supplementary material

Below is the link to the electronic supplementary material.

Supplementary Material 1

## Data Availability

The data supporting the results of this study can be used by anyone through the 2017 & 2020 National Survey of Disabled Persons repository of South Korea. Data can be downloaded by revealing researcher’s information and data use purpose and agreeing to comply with management regulations and requesting permission to use the data.

## Abbreviations

ADL: Activities of daily living
COVID-19: Coronavirus disease 2019
IADL: Instrumental activities of daily living
IDA: The International Disability Alliance
OHCHR: The Office of the High Commissioner for Human Rights
SACH: Self-assessed changes in health
