# Supplementary material for: A comparison of factors associated with unmet healthcare needs in people with disabilities before and after COVID-19: a nationally representative population-based study
Source: BMC Health Serv Res. 2024 Jan 24;24:134. doi: 10.1186/s12913-024-10579-y (PMC10809632; doi:10.1186/s12913-024-10579-y)
Supplement: Supplementary file 1 — Supplementary Material 1 [file 12913_2024_10579_MOESM1_ESM.docx]

Appendix 1. Characteristics before and after COVID-19

| Characteristics | | Categories or range | Before COVID-19 outbreak  (n = 1,884) | After COVID-19 outbreak  (n = 1,884) |
| --- | --- | --- | --- | --- |
|  |  |  | n (%) or Mean±SD | |
| Unmet healthcare needs | | Yes | 360 (19.1) | 604 (32.1) |
|  |  | No | 1,524 (80.9) | 1,280 (67.9) |
| Predisposing  factors | Sex | Male | 1,084 (57.5) | 1,105 (58.7) |
|  |  | Female | 800 (42.5) | 779 (41.3) |
|  | Age (years) |  | 60.84 ± 16.31 | 60.46 ± 16.03 |
|  | Spouse | Yes | 962 (51.1) | 964 (51.2) |
|  |  | No^a^ | 922 (48.9) | 920 (48.8) |
|  | Education | ≤Elementary school | 712 (37.8) | 715 (38.0) |
|  |  | Middle school | 352 (18.7) | 338 (17.9) |
|  |  | High school | 590 (31.3) | 584 (31.0) |
|  |  | ≥College | 230 (12.2) | 247 (13.1) |
| Enabling factors | Type of disability | Physical disability | 1,569 (83.3) | 1,573 (83.5) |
|  |  | Intellectual disability | 315 (16.7) | 311 (16.5) |
|  | Degree of disability | Mild (grade 4–6) | 1,109 (58.9) | 1,110 (58.9) |
|  |  | Severe (grade 1–3) | 775 (41.1) | 774 (41.1) |
|  | Residential area | Seoul | 170 (9.0) | 191 (10.1) |
|  |  | Metropolitan | 511 (27.1) | 509 (27.0) |
|  |  | Others^b^ | 1,203 (63.9) | 1,184 (62.8) |
|  | Monthly income (10,000 KRW) | | 220.12 ± 186.64 | 204.16 ± 194.02 |
|  | Employment status | Employed | 761 (40.4) | 649 (34.4) |
|  |  | Unemployed | 1,123 (59.6) | 1,235 (65.6) |
|  | National Basic Livelihood | Beneficiary | 376 (20.0) | 520 (27.6) |
|  |  | Non-beneficiary | 1,508 (80.0) | 1,364 (72.4) |
|  | ADL | 1–4^c^ | 1.16 ± 0.37 | 1.20 ± 0.52 |
|  | IADL | 1–4^c^ | 1.44 ± 0.69 | 1.49 ± 0.81 |
|  | Going outdoors independently | Yes | 1,658 (88.0) | 1,552 (82.4) |
|  |  | No | 226 (12.0) | 332 (17.6) |
|  | Owned a car | Yes | 923 (49.0) | 948 (50.3) |
|  |  | No | 961 (51.0) | 936 (49.7) |
|  | Experience of discrimination | 1–4^d^ | 1.90 ± 0.78 | 2.15 ± 0.70 |
|  | Satisfaction with medical staff’s understanding of disability | 1–5^e^ | 3.51 ± 0.87 | 3.60 ± 0.70 |
|  | Satisfaction with communication with medical staff | 1–5^e^ | 3.65 ± 0.68 | 3.66 ± 0.70 |
|  | Satisfaction with medical institution facilities and equipment | 1–5^e^ | 3.50 ± 0.71 | 3.56 ± 0.65 |
| Need  factors | Subjective health status | 1–5^f^ | 3.39 ± 0.90 | 3.40 ± 0.90 |
|  | Chronic disease | Yes | 1,438 (76.3) | 1,256 (66.7) |
|  |  | No | 446 (23.7) | 628 (33.3) |
|  | Number of chronic diseases | 1 | 404 (21.4) | 551 (29.2) |
|  |  | ≥2 | 1,034 (54.9) | 705 (37.4) |
|  | Depressive symptoms | Yes | 276 (14.6) | 302 (16.0) |
|  |  | No | 1,608 (85.4) | 1,582 (84.0) |
|  | Regular medical care | Yes | 1,481 (78.6) | 1,369 (72.7) |
|  |  | No | 403 (21.4) | 515 (27.3) |
| *Note.* n, %=unweighted; KRW=Korean won; SD=Standard Deviation; ^a^No spouse= Widowed, Divorced, Separated, or Never married; ^b^Others=Sejong-si, 8 provinces, or Jeju-do; ^c^High scores indicate that more support is needed for ADL or IADL; ^d^High scores indicate more experiences of discrimination; ^e^High scores indicate better satisfaction; ^f^High scores indicate worse subjective health status | | | | |

Appendix 2. Factors associated with unmet healthcare needs in people with physical disabilities before and after COVID-19

| Variables | | Categories | Before COVID-19 | | | | | After COVID-19 | | | | |  |
| --- | --- | --- | --- | --- | --- | --- | --- | --- | --- | --- | --- | --- | --- |
|  |  |  | B | S.E. | OR | 95% CI | *p* | B | S.E. | OR | 95% CI | *p* |  |
| **Predisposing Factors** | | |  |  |  |  |  |  |  |  |  |  |  |
| Sex | Female  (ref. Male) | | 0.441 | 0.155 | 1.555 | 1.146–2.108 | 0.005 | 0.135 | 0.131 | 1.144 | 0.885–1.481 | 0.305 |  |
| Age | | | –0.016 | 0.008 | 0.984 | 0.969–0.999 | 0.034 | –0.008 | 0.006 | 0.992 | 0.979–1.004 | 0.190 |  |
| Spouse | No  (ref. Yes) | | 0.055 | 0.15 | 1.056 | 0.786–1.418 | 0.717 | 0.061 | 0.129 | 1.063 | 0.825–1.370 | 0.636 |  |
| Education | ≤Elementary school | | –0.185 | 0.282 | 0.831 | 0.479–1.443 | 0.511 | 0.217 | 0.231 | 1.242 | 0.790–1.954 | 0.348 |  |
|  | Middle school | | 0.144 | 0.268 | 1.155 | 0.683–1.955 | 0.591 | 0.031 | 0.229 | 1.031 | 0.659–1.615 | 0.893 |  |
|  | High school  (ref. ≥College) | | –0.261 | 0.249 | 0.77 | 0.472–1.256 | 0.296 | –0.109 | 0.203 | 0.896 | 0.602–1.335 | 0.590 |  |
| **Enabling Factors** | | |  |  |  |  |  |  |  |  |  |  |  |
| Degree of disability | Severe  (ref. Mild) | | –0.16 | 0.162 | 0.852 | 0.620–1.171 | 0.324 | 0.023 | 0.139 | 1.023 | 0.779–1.343 | 0.870 |  |
| Monthly household income | | | 0 | 0 | 1 | 0.999–1.001 | 0.594 | 0.000 | 0.000 | 1.000 | 0.999–1.001 | 0.506 |  |
| Employment status | Employed  (ref. Unemployed) | | 0.154 | 0.168 | 1.166 | 0.839–1.621 | 0.359 | 0.165 | 0.147 | 1.179 | 0.883–1.574 | 0.264 |  |
| National basic livelihood | Beneficiary  (ref. Non-beneficiary) | | –0.371 | 0.21 | 0.69 | 0.458–1.041 | 0.077 | –0.048 | 0.154 | 0.953 | 0.704–1.289 | 0.753 |  |
| ADL dependency | | | –0.013 | 0.025 | 0.987 | 0.940–1.037 | 0.599 | 0.009 | 0.016 | 1.009 | 0.978–1.041 | 0.574 |  |
| IADL dependency | | | 0.081 | 0.03 | 1.084 | 1.022–1.150 | 0.007 | 0.046 | 0.020 | 1.047 | 1.006–1.090 | 0.025 |  |
| Going outdoors independently | Yes  (ref. No) | | 0.405 | 0.328 | 1.499 | 0.788–2.849 | 0.217 | 0.181 | 0.219 | 1.198 | 0.779–1.842 | 0.411 |  |
| Owned a car | Yes  (ref. No) | | –0.311 | 0.167 | 0.732 | 0.528–1.016 | 0.063 | –0.109 | 0.141 | 0.897 | 0.680–1.182 | 0.439 |  |
| Experience of discrimination | | | –0.091 | 0.092 | 0.913 | 0.762–1.094 | 0.323 | 0.365 | 0.137 | 1.440 | 0.101–1.883 | 0.008 |  |
| Satisfaction with medical staff’s understanding of disability | | | –0.134 | 0.094 | 0.874 | 0.727–1.051 | 0.154 | 0.077 | 0.117 | 1.080 | 0.859–1.358 | 0.509 |  |
| Satisfaction with communication with medical staff | | | 0.074 | 0.122 | 1.076 | 0.847–1.368 | 0.547 | 0.031 | 0.122 | 1.031 | 0.812–1.309 | 0.802 |  |
| Satisfaction with medical institution facilities and equipment | | | –0.252 | 0.105 | 0.777 | 0.633–0.954 | 0.016 | –0.167 | 0.106 | 0.846 | 0.687–1.042 | 0.116 |  |
| **Need Factors** | | |  |  |  |  |  |  |  |  |  |  |  |
| Subjective health status | | | –0.59 | 0.101 | 0.554 | 0.454–0.676 | <0.001 | –0.161 | 0.080 | 0.851 | 0.728-0.996 | 0.044 |  |
| Chronic disease | Yes  (ref. No) | | 0.213 | 0.256 | 1.238 | 0.749–2.045 | 0.405 | 0.985 | 0.176 | 2.677 | 1.895-3.783 | <0.001 |  |
| Depressive symptom | Yes  (ref. No) | | 0.558 | 0.17 | 1.747 | 1.252–2.438 | 0.001 | 0.797 | 0.150 | 2.220 | 1.654-2.980 | <0.001 |  |
| Regular medical care | Yes  (ref. No) | | –0.329 | 0.246 | 0.72 | 0.445–1.165 | 0.181 | –0.598 | 0.173 | 0.550 | 0.392-0.772 | 0.001 |  |
| **-2 Log likelihood** | | | **1404.497** | | | | | **1824.361** | | | | |  |
| **Nagelkerke R²** | | | **0.140** | | | | | **0.144** | | | | |  |
| **Hosmer–Lemeshow test** | | | **χ² = 6.500, *p* = 0.591** | | | | | **χ ² = 2.956, *p* = 0.937** | | | | |  |
| *Note.* ADL=activities of daily living; B=unstandardized coefficient; CI=confidence interval; COVID=corona virus disease; IADL= instrumental activities of daily living; OR=odds ratio; ref.=reference; S.E.=standard error | | | | | | | | | | | | |  |

Appendix 3. Factors associated with unmet healthcare needs in people with intellectual disabilities before and after COVID-19

| Variables | | Categories | Before COVID-19 | | | | | After COVID-19 | | | | |  |
| --- | --- | --- | --- | --- | --- | --- | --- | --- | --- | --- | --- | --- | --- |
|  |  |  | B | S.E. | OR | 95% CI | *p* | B | S.E. | OR | 95% CI | *p* |  |
| **Predisposing Factors** | | |  |  |  |  |  |  |  |  |  |  |  |
| Sex | Female  (ref. Male) | | 0.089 | 0.33 | 1.093 | 0.572–2.089 | 0.787 | 0.459 | 0.315 | 1.582 | 0.853–2.932 | 0.145 |  |
| Age | | | 0.026 | 0.017 | 1.027 | 0.992–1.062 | 0.134 | 0.020 | 0.017 | 1.021 | 0.988–1.055 | 0.219 |  |
| Spouse | No  (ref. Yes) | | –0.141 | 0.469 | 0.868 | 0.347–2.176 | 0.763 | 0.271 | 0.445 | 1.311 | 0.548–3.138 | 0.542 |  |
| Education | ≤Elementary school | | 0.193 | 0.96 | 1.213 | 0.185–7.963 | 0.841 | 0.834 | 0.815 | 2.304 | 0.466–11.376 | 0.306 |  |
|  | Middle school | | –0.231 | 1.01 | 0.794 | 0.110–5.750 | 0.819 | 1.254 | 0.827 | 3.506 | 0.693–17.731 | 0.129 |  |
|  | High school  (ref. ≥College) | | 0.871 | 0.828 | 2.39 | 0.472–12.111 | 0.293 | 0.991 | 0.711 | 2.694 | 0.669–10.850 | 0.163 |  |
| **Enabling Factors** | | |  |  |  |  |  |  |  |  |  |  |  |
| Monthly household income | | | –0.003 | 0.002 | 0.997 | 0.994–1.000 | 0.060 | 0.002 | 0.001 | 1.002 | 1.000–1.004 | 0.084 |  |
| Employment status | Yes  (ref. No) | | –0.398 | 0.425 | 0.672 | 0.292–1.544 | 0.349 | –0.100 | 0.388 | 0.905 | 0.423–1.937 | 0.798 |  |
| National basic livelihood | Beneficiary  (ref. Non-beneficiary) | | 0.062 | 0.369 | 1.064 | 0.516–2.193 | 0.866 | 0.023 | 0.346 | 1.023 | 0.519–2.018 | 0.947 |  |
| ADL dependency | | | –0.018 | 0.059 | 0.982 | 0.875–1.103 | 0.763 | –0.061 | 0.031 | 0.941 | 0.885–1.001 | 0.053 |  |
| IADL dependency | | | 0.068 | 0.037 | 1.070 | 0.995–1.150 | 0.067 | 0.040 | 0.025 | 1.041 | 0.991–1.093 | 0.113 |  |
| Going outdoors independently | Yes  (ref. No) | | 0.428 | 0.433 | 1.534 | 0.656–3.585 | 0.324 | 0.363 | 0.399 | 1.437 | 0.658–3.142 | 0.363 |  |
| Owned a car | Yes  (ref. No) | | –0.019 | 0.410 | 0.982 | 0.439–2.192 | 0.964 | –0.283 | 0.388 | 0.753 | 0.352–1.613 | 0.466 |  |
| Experience of discrimination | | | –0.703 | 0.259 | 0.495 | 0.298–0.823 | 0.007 | 0.432 | 0.213 | 1.540 | 1.015–2.338 | 0.420 |  |
| Satisfaction with medical staff’s understanding of disability | | | –0.193 | 0.238 | 0.824 | 0.517–1.316 | 0.418 | –0.145 | 0.231 | 0.865 | 0.550–1.359 | 0.530 |  |
| Satisfaction with communication with medical staff | | | –0.318 | 0.314 | 0.727 | 0.393–1.346 | 0.311 | 0.208 | 0.210 | 1.231 | 0.815–1.860 | 0.323 |  |
| Satisfaction with medical institution facilities and equipment | | | 0.183 | 0.292 | 1.201 | 0.678–2.127 | 0.530 | -0.141 | 0.246 | 0.868 | 0.536–1.405 | 0.565 |  |
| **Need Factors** | | |  |  |  |  |  |  |  |  |  |  |  |
| Subjective health status | | | –0.106 | 0.216 | 0.899 | 0.589–1.373 | 0.623 | –0.133 | 0.192 | 0.875 | 0.601–1.275 | 0.488 |  |
| Chronic disease | Yes  (ref. No) | | –0.535 | 0.460 | 0.586 | 0.238–1.441 | 0.244 | 1.624 | 0.394 | 5.075 | 2.344–10.986 | <0.001 |  |
| Depressive symptom | Yes  (ref. No) | | –0.011 | 0.576 | 0.989 | 0.320–3.057 | 0.984 | 0.486 | 0.460 | 1.626 | 0.659–4.007 | 0.291 |  |
| Regular medical care | Yes  (ref. No) | | –0.163 | 0.452 | 0.850 | 0.351–2.060 | 0.719 | –1.236 | 0.411 | 0.290 | 0.130–0.650 | 0.003 |  |
| **-2 Log likelihood** | | | **257.647** | | | | | **313.890** | | | | |  |
| **Nagelkerke R²** | | | **0.156** | | | | | **0.212** | | | | |  |
| **Hosmer–Lemeshow test** | | | **χ² = 4.147, *p*=0.844** | | | | | **χ² = 8.784, *p* = 0.361** | | | | |  |
| *Note.* ADL=activities of daily living; B=unstandardized coefficient; CI=confidence interval; COVID=corona virus disease; IADL= instrumental activities of daily living; OR=odds ratio; ref.=reference; S.E.=standard error | | | | | | | | | | | | |  |
